# QTL mapping and GWAS reveal candidate genes controlling capsaicinoid content in *Capsicum*

**DOI:** 10.1111/pbi.12894

**Published:** 2018-03-02

**Authors:** Koeun Han, Hea‐Young Lee, Na‐Young Ro, On‐Sook Hur, Joung‐Ho Lee, Jin‐Kyung Kwon, Byoung‐Cheorl Kang

**Affiliations:** ^1^ Department of Plant Science Plant Genomics and Breeding Institute Vegetable Breeding Research Center College of Agriculture and Life Sciences Seoul National University Seoul Korea; ^2^ National Academy of Agricultural Science Rural Development Administration Jeonju Korea

**Keywords:** capsaicin, dihydrocapsaicin, genomewide association study (GWAS), pepper, pungency, quantitative trait locus (QTL)

## Abstract

Capsaicinoids are unique compounds produced only in peppers (*Capsicum* spp.). Several studies using classical quantitative trait loci (QTLs) mapping and genomewide association studies (GWAS) have identified QTLs controlling capsaicinoid content in peppers; however, neither the QTLs common to each population nor the candidate genes underlying them have been identified due to the limitations of each approach used. Here, we performed QTL mapping and GWAS for capsaicinoid content in peppers using two recombinant inbred line (RIL) populations and one GWAS population. Whole‐genome resequencing and genotyping by sequencing (GBS) were used to construct high‐density single nucleotide polymorphism (SNP) maps. Five QTL regions on chromosomes 1, 2, 3, 4 and 10 were commonly identified in both RIL populations over multiple locations and years. Furthermore, a total of 109 610 SNPs derived from two GBS libraries were used to analyse the GWAS population consisting of 208 *C. annuum*‐clade accessions. A total of 69 QTL regions were identified from the GWAS, 10 of which were co‐located with the QTLs identified from the two biparental populations. Within these regions, we were able to identify five candidate genes known to be involved in capsaicinoid biosynthesis. Our results demonstrate that QTL mapping and GBS‐GWAS represent a powerful combined approach for the identification of loci controlling complex traits.

## Introduction

Hot peppers (*Capsicum* spp.) contain capsaicinoids, unique compounds that produce a burning sensation called pungency. Capsaicinoids are believed to protect pepper fruits from diseases, such as *Fusarium* (Tewksbury *et al*., 2008), and enable the dispersal of their seeds by birds, which, unlike mammals, cannot detect the pungency and do not harm the seeds (Tewksbury and Nabhan, 2001). Humans use pungent peppers as a vegetable, in sauces and in food additives (Aza‐Gonzalez *et al*., 2011), while capsaicinoids are also used in pharmaceuticals and other medicines (Aza‐Gonzalez *et al*., 2011; Luo *et al*., 2011).

The presence of capsaicinoids is mainly controlled by *Pun1*, which encodes capsaicin synthase (CS) (Stewart *et al*., 2005). CS functions in the final step of capsaicinoid biosynthesis, and the expression of *Pun1* is detected only in the fruits (Stewart *et al*., 2005, 2007). Most nonpungent pepper cultivars have nonfunctional *Pun1* alleles, containing a deletion (*pun1*), a frameshift mutation (*pun1* ^*2*^) or an early stop codon (*pun1* ^*3*^) (Stellari *et al*., 2010; Stewart *et al*., 2005, 2007). Mutations in another gene, *Putative Aminotransferase* (*pAMT*), convert biosynthesis of capsaicinoids into that of capsinoids, which are about 1000 times less pungent than capsaicinoids (Lang *et al*., 2009). Several nonfunctional *pamt* alleles have been identified and used to breed high‐capsinoid pepper varieties (Jang *et al*., 2015; Jeong *et al*., 2015; Tanaka *et al*., 2014, 2015). Gene expression analyses have revealed other genes that function in the capsaicinoid biosynthesis pathway, including those encoding phenylalanine ammonia lyase (*Pal*), 3‐keto‐acyl‐ACP synthase (*Kas*) and thioesterase (*Fat*) (Curry *et al*., 1999; Aluru *et al*., 2003; Kim, 2001). The expression levels of these genes correlate with the capsaicinoid content, but allelic variations affecting capsaicinoid biosynthesis have been identified only for *Pun1* and *pAMT* (Koeda *et al*., 2015).

The capsaicinoid content of peppers is controlled by quantitative trait loci (QTLs) (Collins *et al*., 1995; Sanatombi and Sharma, 2008), which have been identified from several interspecific populations (Ben‐Chaim *et al*., 2006; Blum *et al*., 2003; Lee *et al*., 2016b; Yarnes *et al*., 2013). However, direct comparisons between the QTLs from different studies are not possible due to the limited numbers of common markers between populations and the low density of genetic maps. Although capsaicinoid biosynthesis genes may be located at these QTLs, no likely candidate genes underlying the QTLs have been proposed in these previous studies.

Traditional QTL mapping is highly dependent on the genetic diversity of the two parents, and the effects of the detected QTLs can vary between populations. QTL regions can also be quite large, incorporating too many genes to investigate as potential candidate genes. The limitations of QTL analysis can be overcome using genomewide association studies (GWAS), which can narrow down the candidate regions using natural populations. GWAS does have the potential for false‐positive error however, and validation of the results is necessary (Korte and Farlow, 2013; Zhu *et al*., 2008). The number of markers used in the GWAS highly affects its results. Genotyping by sequencing (GBS) is one of the genotyping methods used for GWAS, and GBS‐GWAS approaches have been successfully applied to the identification of candidate genes controlling quantitative traits in plant species including soya bean (*Glycine max*), diploid alfalfa (*Medicago sativa*), chickpea (*Cicer arietinum*) and maize (*Zea mays*) (Navarro *et al*., 2017; Sakiroglu and Brummer, 2017; Sonah *et al*., 2015; Upadhyaya *et al*., 2016). There have been only two reports on the use of GWAS for analysis of capsaicinoid content in *Capsicum*. Using 176 simple sequence repeats and 96 *C. annuum* accessions, Nimmakayala *et al*. (2014) identified one marker on chromosome 1 that was associated with the capsaicin and dihydrocapsaicin contents, while Nimmakayala *et al*. (2016) used 7331 single nucleotide polymorphisms (SNPs), of which 72 were found to be associated with capsaicinoid content, including in a candidate gene encoding an ankyrin‐like protein which has acyltransferase function similar to CS.

Genomewide association study can have high rates of false‐positive errors due to the population structures (Zhu *et al*., 2008). The combination of GWAS and QTL analyses can compensate for the limitations of each approach, enabling the identification of loci controlling agronomically important quantitative traits. Such combined approaches have been successfully used to identify candidate genes controlling flowering time, panicle architecture, leaf architecture, frost resistance and seed‐related traits in *Arabidopsis thaliana*, rice (*Oryza sativa*), maize, winter faba bean (*Vicia faba*) and soya bean, respectively (Brachi *et al*., 2010; Crowell *et al*., 2016; Sallam *et al*., 2016; Sonah *et al*., 2015; Tian *et al*., 2011).

In this study, we performed QTL mapping in one intraspecific and one interspecific RIL population of *Capsicum*. High‐density genetic maps and phenotype data from multiple environments were used to ensure an accurate linkage analysis. In addition, a total of 208 *C. annuum*‐clade accessions, including *C. annuum*,* C. chinense* and *C. frutescens*, were genotyped by GBS and analysed using GWAS. By comparing the physical locations of the QTLs identified in this study and previous work, five candidate genes in the capsaicinoid biosynthesis pathway were proposed.

## Results

### Measurement of capsaicinoid content in the biparental populations

**Figure 1:**
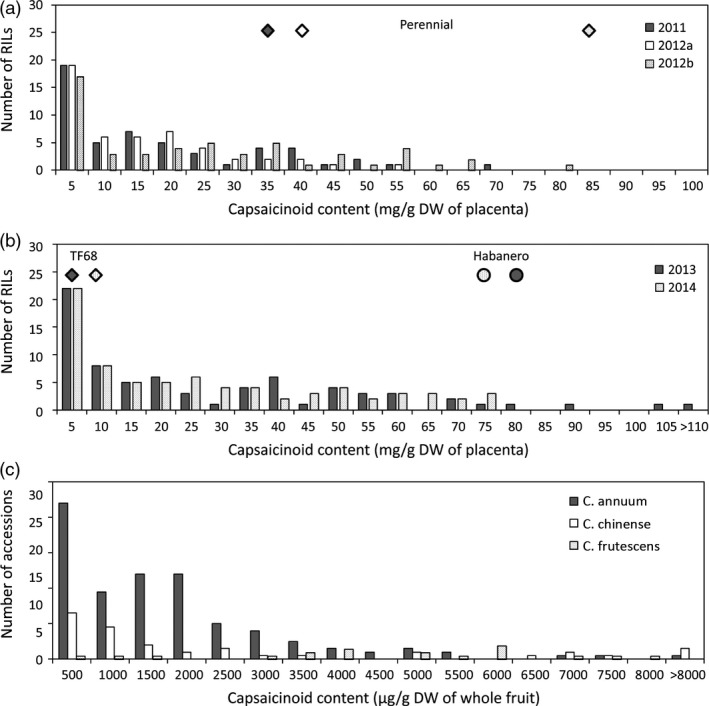
Capsaicinoid contents of the ‘PD’ RILs (a), ‘TH’ RILs (b) and GWAS population (c). Diamonds and circles show the average contents of the maternal parents (Perennial or TF68) and the paternal parent (Habanero), respectively. Dempsey, the paternal parent of ‘PD’ RILs, was nonpungent.

‘Perennial’ is a pungent small pepper line, while ‘Dempsey’ is a nonpungent bell pepper cultivar. Due to the nonfunctional *pun1* allele of the paternal line ‘Dempsey’, the ‘PD’ RIL population created in this cross had a 1:1 segregation ratio of pungency, comprising 56 pungent and 64 nonpungent RILs. Capsaicinoid content was evaluated from plants grown in three different environments. The capsaicinoid contents in the placenta of fruits from the pungent parent ‘Perennial’ grown in Anseong were 38 013 and 31 518 μg/g dry weight (DW) in 2011 and 2012a, whereas the capsaicinoid content of placental tissues from plants grown in Suwon (2012b) was 81 257 μg/g DW (Table S1). The average capsaicinoid contents of the placental tissues from the pungent RILs were 16 555, 13 005 and 22 058 μg/g DW in 2011, 2012a and 2012b, respectively (Figure 1a; Table S1). Transgressive segregation was observed in 2011 and 2012a.

The parents of the ‘TH’ RILs, ‘Habanero’ and ‘TF68’, are both pungent, with ‘Habanero’ found to be more pungent than ‘TF68’ (Figure 1b; Table S2). The capsaicinoid contents of the placental tissues in ‘TF68’ and ‘Habanero’ were 5672 and 89 825 μg/g DW, respectively, in 2013, and 7199 and 73 819 μg/g DW, respectively, in 2014. The average placental tissue capsaicinoid contents of the 85 ‘TH’ RILs phenotyped for QTL mapping were 25 809 and 23 953 μg/g DW in 2013 and 2014, respectively. In both years, RILs more pungent than ‘Habanero’ were identified.

The distribution of total capsaicinoid content showed a positive skew in both the ‘PD’ RIL and ‘TH’ RIL populations. A large percentage of the ‘TH’ RILs had a lower or similar capsaicinoid content compared with ‘TF68’, and these skewed distributions were also detected for the individual capsaicin and dihydrocapsaicin contents (data not shown). The contents of capsaicin, dihydrocapsaicin and the total capsaicinoids showed a high level of correlation between all environments, with Pearson correlation coefficients of between 0.64 and 0.99 (Figure S1).

### Bin map of biparental populations

**Figure 2:**
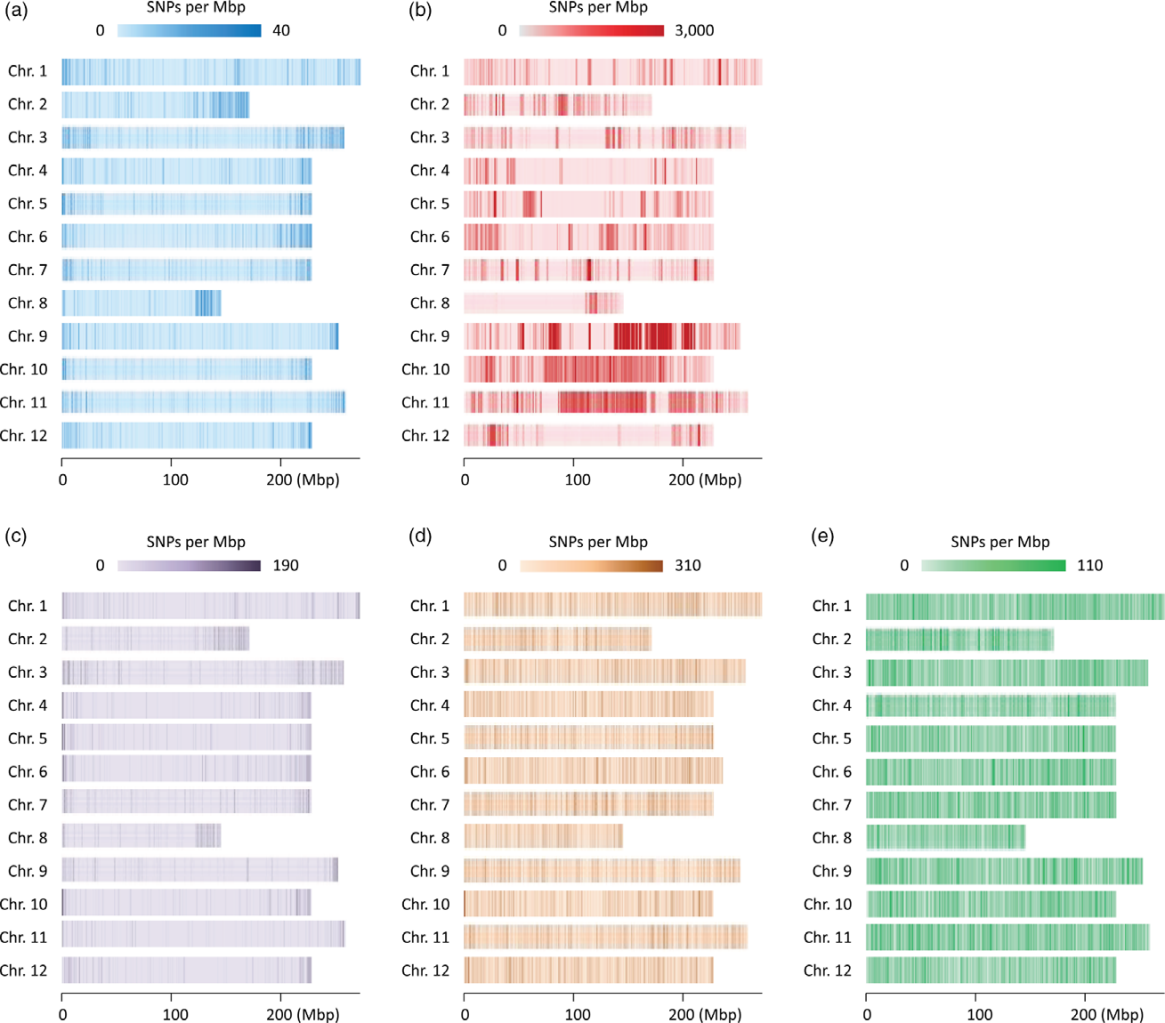
Single nucleotide polymorphism (SNP) density (number of SNPs per Mbp) of the ‘TH’ (a) and ‘PD’ (b) RIL populations, as well as the GWAS populations digested using PstI/MseI (c), EcoRI/MseI (d) and PstI/MseI and EcoRI/MseI (e).

Genotypes of ‘TH’ RILs were analysed using GBS after the preparation of libraries from PstI/MseI‐digested DNA. The average number of reads per sample was around 4 million, and a total of 8587 SNPs were detected by aligning the sequences obtained from GBS to the *C. annuum* ‘CM334’ reference genome (Table 1). The SNPs were more densely distributed at the ends of the chromosomes (Figure 2a). To correct for missing data and genotyping error, a sliding window approach was used (Han *et al*., 2016). Recombination breakpoints were determined using 18 consecutive SNPs as one sliding window, and a high‐density bin map of the ‘TH’ RIL population was constructed. The map consisted of 1089 bins with an average genetic distance of 1.0 cm (Table S3; Table S4). Among the 12 linkage groups, the genetic distance of chromosome 1 was longest and chromosome 8 was shortest.

**Table 1:** Number of sequencing reads and SNPs used for GWAS and QTL mapping

	TH RIL	PD RIL	GWAS population
# of accessions (lines)	85	56	208
Genotyping method	GBS (PstI/MseI)	WGS	GBS (PstI/MseI and EcoRI/MseI)
Avg. # of reads per sample	4 103 757	15 738 890^a^	3 326 422
Total # of SNPs	8587	1 431 214	109 610
Avg. distance between SNPs (bp)	328 662	2713	25 093

a Number of sites sequenced by paired‐end reads.

GBS, genotyping by sequencing; WGS, whole‐genome resequencing.

For the genotyping of the ‘PD’ RIL population, previously reported whole‐genome resequencing data were used (Han *et al*., 2016). Due to the higher density of SNPs and larger number of RILs in ‘PD’ than ‘TH’, more recombination breakpoints were identified in ‘PD’ RILs (3983) than in ‘TH’ RILs (2386); therefore, the average distance between bins was shorter in ‘PD’ RILs (0.5 cm). The total genetic lengths of the ‘PD’ RIL and ‘TH’ RIL maps were estimated to be 1372 and 1127 cm, respectively. The two bin maps were compared with the *C. annuum* ‘CM334’ reference genome, and the physical position of each bin was determined (Figure S2). With the exception of chromosome 8, the overall genetic and physical positions of the bins were colinear. More bins were detected in the middle of chromosomes for ‘PD’ than in ‘TH’, which reflected the distribution of the SNPs (Figure 2b).

### QTL mapping for capsaicinoid content

Quantitative trait locus controlling the contents of capsaicin, dihydrocapsaicin and total capsaicinoid were detected in ‘PD’ RILs and ‘TH’ RILs (Table 2). For ‘PD’, the capsaicinoid contents of 56 RILs and an ultra‐high‐density bin map of 120 RILs were used to identify 5, 9 and 8 QTLs for the capsaicin, dihydrocapsaicin and total capsaicinoid contents, respectively. The QTLs were located on chromosomes 1, 2, 3, 4, 6, 10 and 12. Five QTLs, *PD‐cap10* (capsaicin‐related), *PD‐dicap1.1* and *PD‐dicap10.2* (dihydrocapsaicin‐related), and *PD‐total2* and *PD‐total10.2* (total capsaicinoid‐related) were independently identified in plants grown in two environments. Moreover, with the exception of five QTLs, the majority of the QTL regions corresponded to more than two traits, such as the region containing *PD‐cap10*,* PD‐dicap10.2* and *PD‐total10*. RILs with the ‘Perennial’ SNP genotypes at the QTLs showed an increased capsaicinoid level, except for those at *PD‐dicap2.1*,* PD‐dicap12* and *PD‐total12*. *PD‐dicap1.1* showed the highest LOD value (8.7) and *PD‐dicap10.2* showed the highest *R* ^2^ value (28.8%).

**Table 2:** Quantitative trait loci (QTLs) controlling capsaicin, dihydrocapsaicin and total capsaicinoid contents detected in two RILs

Population	Trait	QTL	Year	Chr.	Location (cM)	LOD	R^2^ (%)	Direction^a^	Additive effect
PD RIL	CAP	***PD‐cap1***	2011	1	50.4–52.2	7.0	25.3	+	5.0
		*PD‐cap2*	2012a	2	83.5–87.8	5.0	18.4	+	3.5
		***PD‐cap3***	2012b	3	82.7–90.2	5.0	14.4	+	4.9
		*PD‐cap6*	2012b	6	47.3–50.5	7.0	22.3	+	5.3
		***PD‐cap10***	2011, 2012a	10	28.6–35.4	4.6–6.6	15.2–25.2	+	3.6–3.9
	DICAP	***PD‐dicap1.1***	2011, 2012b	1	47.1–61.1	4.6–8.7	13.8–27.7	+	3.7–3.9
		*PD‐dicap1.2*	2011	1	117.8–119.5	4.0	11.0	+	2.5
		*PD‐dicap1.3*	2011	1	121.5–128.3	4.0	11.0	+	2.5
		***PD‐dicap2.1***	2012a	2	50.0–56.4	3.0	11.2	−	2.3
		*PD‐dicap2.2*	2012a	2	82.8–87.1	5.0	15.5	+	3.0
		*PD‐dicap4*	2011	4	45.4–50.3	5.0	13.0	+	2.8
		*PD‐dicap10.1*	2011	10	18.8–26.1	5.0	15.2	+	2.9
		***PD‐dicap10.2***	2012a, 2012b	10	29.1–34.6	6.6–7.3	21.2–28.8	+	3.6–4.6
		*PD‐dicap12*	2012a	12	28.4–32.6	3.0	11.1	−	2.4
	TOTAL	***PD‐total1.1***	2011	1	50.0–60.5	3.0	11.1	+	8.8
		*PD‐total1.2*	2011	1	122.3–128.8	4.0	10.2	+	5.4
		*PD‐total2*	2012a, 2012b	2	83.2–88.0	4.0–5.2	15.5–17.1	+	6.5–10.5
		*PD‐total4.1*	2011	4	42.7–49.2	4.0	10.7	+	5.8
		***PD‐total4.2***	2012b	4	67.3–72.5	4.0	15.2	+	8.2
		*PD‐total10.1*	2011	10	22.1–24.9	5.0	14.8	+	6.8
		***PD‐total10.2***	2011, 2012a	10	28.6–32.8	4.9–7.1	15.7–27.2	+	6.9–7.3
		*PD‐total12*	2012a	12	28.4–32.5	4.0	11.6	−	5.0
TH RIL	CAP	*TH‐cap1.1*	2014	1	5.6–6.6	4.1	8.4	−	5.1
		*TH‐cap1.2*	2014	1	9.6–12.6	4.5	9.1	−	5.0
		*TH‐cap1.3*	2014	1	14.5–18.5	7.2	13.7	−	6.3
		***TH‐cap1.4***	2013	1	59.8–61.7	5.4	21.1	−	10.7
		*TH‐cap1.5*	2013, 2014	1	129.2–149.3	4.2–4.6	6.1–11.8	−	3.5–6.7
		*TH‐cap2.1*	2014	2	52.3–56.0	9.8	18.5	−	6.2
		***TH‐cap2.2***	2014	2	58.4–62.0	9.9	18.8	−	6.6
		***TH‐cap4***	2014	4	22.6–29.3	4.7	10.3	+	4.4
	DICAP	*TH‐dicap3.1*	2014	3	57.6–65.7	4.6	9.5	+	4.1
		*TH‐dicap3.2*	2013, 2014	3	65.7–76.0	5.5–5.6	11.2–18.2	+	4.2–7.7
		*TH‐dicap3.3*	2013	3	76.6–81.3	3.6	12.4	+	7.0
		*TH‐dicap6*	2013	6	83.9–86.8	6.0	19.7	−	12.4
		***TH‐dicap10***	2014	10	13.3–27.5	5.2	10.7	−	4.9
	TOTAL	*TH‐total1.1*	2014	1	3.1–6.0	6.2	12.9	−	11.4
		*TH‐total1.2*	2014	1	10.6–13.3	7.9	15.8	−	12.8
		*TH‐total1.3*	2013	1	131.0–139.7	3.8	12.2	−	14.3
		***TH‐total2***	2014	2	57.3–60.8	8.7	16.1	−	12.6
		*TH‐total3.1*	2013	3	65.0–68.1	4.8	14.4	+	11.9
		***TH‐total3.2***	2014	3	86.0–91.7	3.5	6.8	+	8.4
		*TH‐total3.3*	2014	3	94.8–106.1	5.4	10.0	+	10.8
		*TH‐total6*	2013	6	83.9–86.6	3.8	12.9	−	20.7
		***TH‐total10***	2014	10	12.7–22.0	7.8	15.5	−	13.0

a Genotypes that increase the pungency level. +means the genotype resembles that of Perennial or TF68.

Bold QTLs were common to both populations.

CAP, capsaicin; DICAP, dihydrocapsaicin; TOTAL, total capsaicinoid.

The capsaicinoid contents of the ‘TH’ RILs grown in two environments were evaluated for the QTL analysis of this population. A total of 8, 5 and 9 QTLs for the capsaicin, dihydrocapsaicin and total capsaicinoid contents were detected, located on chromosomes 1, 2, 3, 4, 6 and 10 (Table 2). Among these 22 QTLs, 15 had negative additive effects, meaning plants with the ‘Habanero’ genotype showed an increased capsaicinoid content. *TH‐cap2.2* showed the highest LOD score and explained 18.8% of total phenotypic variation of the capsaicin contents. As seen for ‘PD’ RILs, 15 of the ‘TH’ RIL QTLs also controlled two traits; however, no QTL regions were detected to control all three traits.

**Figure 3:**
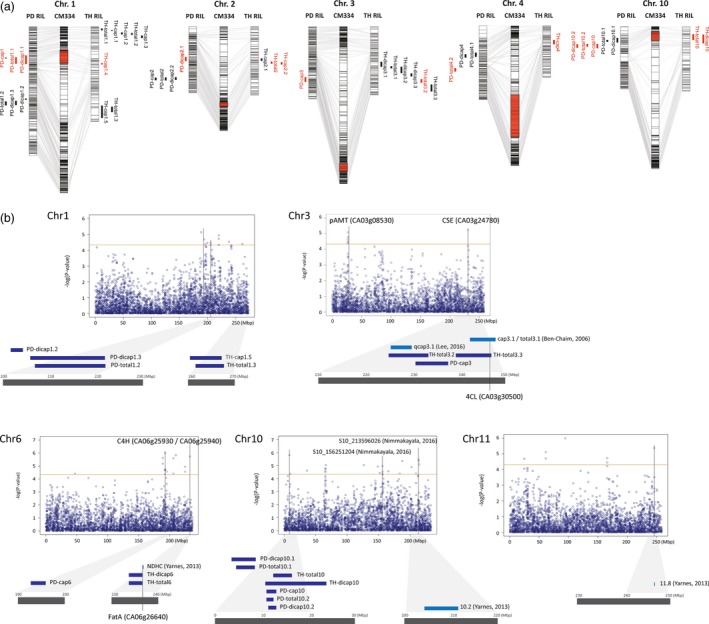
Comparison of QTLs and SNPs associated with capsaicinoid contents. (a) Common QTLs detected from both ‘PD’ and ‘TH’ RIL populations. The QTLs marked in red are common to both populations. (b) Manhattan plot from GWAS and co‐located QTLs. The threshold of the ‐log(*P*) was 4.3. QTLs and genes related to capsaicinoid biosynthesis are indicated.

The physical locations of the QTLs detected in ‘PD’ and ‘TH’ RILs were compared using the *C. annuum* ‘CM334’ reference genome (Figure 3a). Among the 22 QTLs from each population, 9 QTLs from ‘PD’ RILs and 7 from ‘TH’ RILs were located at the same positions. On chromosome 1, *PD‐cap1*,* PD‐dicap1.1*,* PD‐total1.1* and *TH‐cap1.4* were located at 39.6–60.6 Mbp. *PD‐dicap2.1*,* TH‐cap2.2* and *TH‐total2* were located 124.8–132.2 Mbp along chromosome 2, while *PD‐cap3* and *TH‐total3.2* were located 225.1–237.8 Mbp along chromosome 3. *PD‐total4.2* and *TH‐cap4* appeared to be located near the markers of a single region on chromosome 4, but their positions were relatively distant, located at 110.4 and 177.4 Mbp, respectively. The largest numbers of QTLs were located at 9.6–23.9 Mbp along chromosome 10, including *PD‐cap10*,* PD‐dicap10.2*,* PD‐total10.2*,* TH‐dicap10* and *TH‐total10*. The QTLs located on chromosome 10 could explain the contents of capsaicin, dihydrocapsaicin and total capsaicinoids, and the R^2^ value was higher than 15.0%. Five genetic regions on chromosomes 1 (39.6‐60.6 Mbp), 2 (124.8‐132.2 Mbp), 3 (225.1‐237.8 Mbp), 4 (110.4‐177.4 Mbp) and 10 (9.6‐23.9 Mbp) were considered to be common QTLs, and the QTLs located on chromosome 10 were considered the most significant.

### Epistatic control of capsaicinoid content

In both ‘PD’ and ‘TH’ RILs, the distribution graphs of capsaicinoid content showed a positive skew (Figure 1a,b); there were more mildly pungent RILs than extremely pungent RILs. A skewed distribution indicates that there may be epistatic interactions between the QTLs. Using multiple‐interval mapping (MIM), epistatic effects between common QTLs were detected (Table S5; Table S6). In ‘PD’ RILs, additive‐by‐additive epistases between *PD‐cap1* and *PD‐cap10* (capsaicin, 2011), *PD‐dicap2.1* and *PD‐dicap10.2* (dihydrocapsaicin, 2012a), *PD‐dicap1.1* and *PD‐dicap10.2* (dihydrocapsaicin, 2012b) and *PD‐total1.1* and *PD‐total10.2* (total capsaicinoid, 2011) were detected (Table S5). These individual QTLs and the interactions between them could explain 17.5%–45.4% of the variation in their respective capsaicinoid contents.

Additive‐by‐additive epistatic effects between the common QTLs were detected in only one environment (2014) for ‘TH’ RILs, occurring between *TH‐cap2.2* and *TH‐cap4* for capsaicin content, and *TH‐total2*,* TH‐total3.2* and *TH‐total10* for total capsaicinoid content (Table S6). The effects of the individual QTLs (*TH‐total2*,* TH‐total3.2* and *TH‐total10*) and their epistatic effects could explain 57.2% of the total capsaicinoid content variation in 2014.

### SNPs and haplotype blocks of GWAS population

To validate the QTLs detected from the biparental populations, a GWAS study for capsaicinoid content was performed using 208 *C. annuum*‐clade accessions, including 145 from *C. annuum*, 42 from *C. chinense* and 21 from *C. frutescens* (Table S7). The accessions were genotyped using the GBS method; GBS libraries were constructed using two restriction enzyme sets, PstI/MseI and EcoRI/MseI, from which 14 461 and 119 710 significant SNPs were detected, respectively (data not shown). Even distribution of SNPs was detected using EcoRI/MseI than PstI/MseI (Figure 2c,d). After filtering the SNPs for minor allele frequencies and calling rate, a total of 109 610 SNPs were selected for further study (Table 1). In contrast to ‘PD’ and ‘TH’ RILs, the SNPs of the GWAS population were relatively evenly distributed, with an average distance between SNPs of 25 093 bp (Figure 2e). Using these SNPs, the *Capsicum* accessions were divided into three subgroups using a principal component analysis (PCA) (Figure S3a) and a phylogenetic analysis (Figure S3b). These analyses showed that the accessions of the GWAS population were grouped according to their expected species groups, *C. annuum*,* C. chinense* and *C. frutescens*. Eight accessions were not included in any of the subgroups, and four accessions were in different subgroups from the species that was described in their passport data in GenBank where collecting *Capsicum* germplasm. The population structure determined from the PCA was applied for the GWAS.

Haplotype blocks were calculated in each chromosome using less stringent options than the default settings. About 90% of SNPs were grouped into 5513 blocks, and each block contained 3–138 SNPs, with an average of 18 SNPs (Table S8). The block size varied between 3 bp and 2 Mbp, with average block sizes of 567, 438, 547, 395, 465, 526, 434, 225, 506, 505, 535 and 370 kbp for the twelve chromosomes, respectively. The average haplotype block size was 409 kbp, which was larger than the average distance between the SNPs used for the GWAS (Table S8).

### GWAS for capsaicinoid content

For the 208 accessions comprising the GWAS population, the capsaicinoid content was evaluated from freeze‐dried whole fruits. Their total capsaicinoid contents varied from 2 to 16 082 μg/g DW in the whole fruit (Figure 1c), with ten accessions containing less than 10 μg/g DW. Of the 20 accessions with the highest capsaicinoid contents, eight were *C. frutescens*, seven were *C. chinense*, and five accessions were *C. annuum*. The three most pungent accessions, ‘Habanero’, ‘9007’ and ‘Spain 5’, were all accessions of *C. chinense*, which is well known for its pungency (Bosland and Baral, 2007; Bosland *et al*., 2012; Canto‐Flick *et al*., 2008).

We analysed the association of SNPs with the capsaicin, dihydrocapsaicin and total capsaicinoid contents using GWAS. A total of 99 SNPs were associated with capsaicin, 9 were linked to dihydrocapsaicin, and 42 SNPs were associated with the total capsaicinoid content; however, the SNPs associated with the dihydrocapsaicin and total capsaicinoid contents did not exceed the false discovery rate (FDR) threshold, so only the 99 capsaicin‐associated SNPs were considered significant. These 99 SNPs were grouped into 69 genomic regions using a haplotype block estimation (Table S9). Using gene annotation data and SNPs located on haplotype blocks, 213 genes located on 69 associated regions were found and their functions were predicted (Table S9).

Among 69 regions, 10 regions on chromosomes 1, 3, 6 and 10 were co‐located with QTLs detected in the present study (Figure 3b; Table S9), while four regions on chromosomes 10 and 11 were linked to previously detected QTLs and SNPs (Nimmakayala *et al*., 2016; Yarnes *et al*., 2013). On chromosome 1, three regions incorporating six SNPs were co‐located with *PD‐dicap1.3* and *PD‐total1.2*, while another SNP was co‐located with *TH‐cap1.5* and *TH‐total1.3*. One capsaicin‐associated SNP was detected between 230.53 and 231.21 Mbp on chromosome 3, which corresponded to the location of the *TH‐total3.2* and *PD‐cap3* QTLs. On chromosome 6, three regions containing eight SNPs were detected together with *PD‐cap6*. Two regions on chromosome 10, each containing a single SNP, were also validated by QTLs; SNP 10_8241800 was co‐located with *PD‐dicap10.1*,* PD‐total10.1*. And the other SNP 10_9608580 was co‐located with *TH‐total10*,* TH‐dicap10*,* PD‐cap10*,* PD‐dicap10.2* and *PD‐total10.2*. A total of 55 new regions associated with capsaicin contents were detected, with an average –log(*P*) value of 4.84.

### Candidate gene prediction for QTLs controlling capsaicinoid content

From the QTL mapping and GWAS, we were able to identify candidate genes involved in the capsaicinoid biosynthesis pathway. Among the candidate genes from GWAS, two genes expected to function in the capsaicinoid biosynthesis pathway were identified (Table 3; Table S9). *pAMT*, located on chromosome 3, was strongly linked to seven significantly associating SNPs. pAMT mediates the formation of vanillylamine, which is the final step of the phenylpropanoid pathway for the biosynthesis of capsaicinoids (Lang *et al*., 2009); therefore, *pAMT* is a plausible candidate gene for the control of capsaicinoid content. On chromosome 6, *cinnamate 4‐hydroxylase* (*C4H*) was located around 400 kbp away from SNP 6_232803485. C4H is also involved in the phenylpropanoid pathway and has catalytic activity in the biosynthesis of coumarate from cinnamate (Curry *et al*., 1999; Mazourek *et al*., 2009). The comparison of the QTL mapping and GWAS results led to the identification of *caffeoyl shikimate esterase* (*CSE*), located on chromosome 3, as a candidate gene. Two QTLs from this study, one QTL from Lee *et al*. (2016b)and one SNP from the GWAS were linked to *CSE* (Table 3; Figure 3b). Even though the role of *CSE* in the capsaicinoid biosynthesis pathway is unknown, CSE is known to hydrolyse caffeoyl shikimate, which is an intermediate of phenylpropanoid pathway (Vanholme *et al*., 2013). From the QTL mapping results, *TH‐total3.3*, located at 239.4–246.9 Mbp on chromosome 3, was associated with the gene encoding 4‐coumaroyl‐CoA ligase (4CL), which was previously linked to other capsaicinoid QTLs, *cap3.1* and *total3.1* (Ben‐Chaim *et al*., 2006). Another gene, encoding acyl‐ACP thioesterase (*FatA*), functions in the fatty acid biosynthesis pathway and was associated with the QTLs *TH‐dicap6* and *TH‐total6,* as well as QTL *6.8*, which was previously found to be linked to nordihydrocapsaicin content (Yarnes *et al*., 2013). In summary, we propose five candidate genes for the control of capsaicinoid content, *pAMT*,* C4H*,* CSE*,* 4CL* and *FatA*, each with known or potential functions in the capsaicinoid biosynthesis pathway.

**Table 3:** Candidate genes related to capsaicinoid biosynthesis and their associated QTLs or SNPs

Candidate gene (CDS)	PD RIL	TH RIL	GWAS population	Previous study
*pAMT* (CA03g08530)	–	–	3_26745322, 3_26745328, 3_26745367, 3_26770544, 3_26770554, 3_26770560, 3_27438287	–
*C4H* (CA06g25930/CA06g25940)	–	–	6_232803485	–
*CSE* (CA03g24780)	*PD‐cap3*	*TH‐total3.2*	3_230603011	*qcap3.1* (Lee *et al*., 2016b)
*4CL* (CA03g30500)	–	*TH‐total3.3*	–	*cap3.1*,* total3.1* (Ben‐Chaim *et al*., 2006)
*FatA* (CA06g26640)	–	*TH‐dicap6, TH‐total6*	–	*6.8* (Yarnes *et al*., 2013)

**Figure 4:**
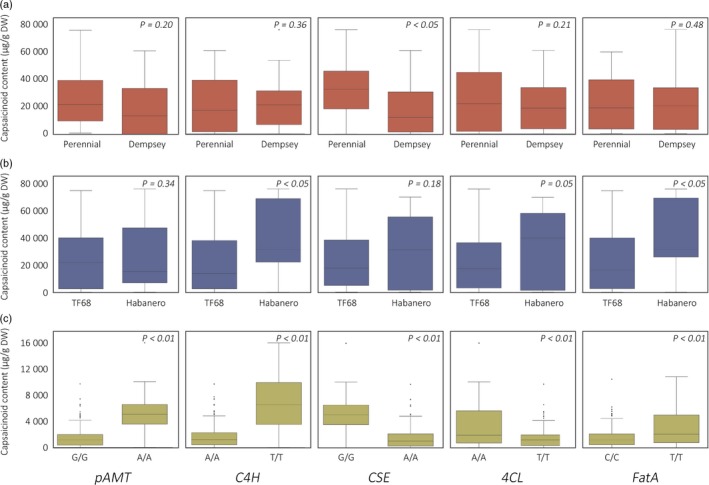
Box plots of capsaicinoid contents regulated by the five candidate genes. Total capsaicinoid contents resulting from the ‘PD’ RIL (a) and ‘TH’ RIL (b) parental genotypes related to each candidate gene in plants grown in 2012b and 2014, respectively. (c) Total capsaicinoid content resulting from reference (‘CM334’) and alternative genotypes related to each candidate gene in plants of the GWAS population. RILs and accessions were classified by the genotype of the most closely linked marker to the target genes. *P*‐values were calculated from an equal variance t‐test for ‘PD’ and ‘TH’ RIL’ and from an unequal variance t‐test for the GWAS population.

To compare the capsaicinoid contents of plants to their genotypes at these candidate genes, individual plants from the RIL and GWAS populations were grouped by their genotypes at *pAMT*,* C4H*,* CSE*,* 4CL* and *FatA*. Bin markers located within 1 Mbp of the candidate genes were used, due to the lack of genotype information for the candidate genes themselves. For ‘PD’ RILs, PD3‐bin56, PD6‐bin174, PD3‐bin200, PD3‐bin216, PD6‐bin179 were used, while TH3‐bin1, TH6‐bin91, TH3‐bin98, TH3‐bin112 and TH6‐bin93 were used for ‘TH’ RILs. For the GWAS population, the genotypes of SNPs 3_26745367, 6_232803485 and 3_230603011, which showed association with capsaicin content, were used to draw box plots for *pAMT*,* C4H* and *CSE*, respectively. The accessions were also separated by their genotypes at SNPs 3_246744919 and 6_234337365, which were not associated with capsaicin content, but were the closest SNPs to *4CL* and *FatA*. In ‘PD’ RILs, only *CSE* was associated with a significant difference in capsaicin content, whereas *C4H*,* FatA* and *4CL* were associated with significant differences in capsaicin content in ‘TH’ RILs (Figure 4). In the GWAS population, differences in the genotypes of all five candidate genes led to highly significant differences in capsaicinoid contents.

## Discussion

### Global comparison of QTLs for capsaicinoid content

Using the *C. annuum* ‘CM334’ reference genome, we compared the physical locations of capsaicinoid‐related QTLs detected in multiple analyses and capsaicinoid content‐associating SNPs from GWAS (Table S10). The QTLs from our research were also compared with those detected in other studies (Ben‐Chaim *et al*., 2006; Blum *et al*., 2003; Lee *et al*., 2016b; Nimmakayala *et al*., 2016; Yarnes *et al*., 2013). Before now, the comparison of QTLs from different studies was not feasible, due to the lack of a reference genome or common markers used for the genetic maps. Here, we used the primer sequences linked to the QTLs to BLAST the ‘CM334’ reference genome (v1.55), or if primer sequences were not publicly available, the closest marker with information was used (Table S10). In total, eight QTLs located on chromosomes 2, 3 and 6 were validated. A shared QTL on chromosome 3, which contains *PD‐cap3* and *TH‐total3.2*, was co‐located with *qcap3.1*, which was previously identified in the F_2_ population derived from a *C. annuum* ‘NB1’ × *C. chinense* ‘Bhut Jolokia’ cross (Lee *et al*., 2016b). *TH‐total3.3* was also linked to *cap3.1* and *total3.1* detected in the *C. annuum* ‘NuMex Rnaky’ × *C. frutescens* ‘BG2814‐6’ F_2:3_ population (Ben‐Chaim *et al*., 2006). *TH‐dicap6* and *TH‐total6* were co‐located with a QTL controlling nordihydrocapsaicin detected in a *C. frutescens* ‘2814‐6’ × *C. annuum* ‘NuMex Rnaky’ RIL (Yarnes *et al*., 2013). The *Pun1* locus was detected from ‘PD’ RILs in the QTLs *PD‐cap2*,* PD‐dicap2.1* and *PD‐total2*. We expected that *Pun1* would be detected due to the segregation of pungency in some RILs having heterozygous loci. Previously, *cap* and *cap7.2*, located on chromosome 7, were detected from two different populations and were thought to constitute a major QTL (Ben‐Chaim *et al*., 2006; Blum *et al*., 2003). This locus was expected to 202‐203 Mbp on chromosome 7 using linked marker information (Table S10), but no QTL or associated SNPs were located in this locus from our QTL or GWAS analyses. Ben‐Chaim *et al*. and Blum and colleagues used *C. frutescens* as a parent to develop interspecific populations. The genetic diversity of this species is unlikely to be represented in our intraspecific population derived from *C. annuum* (‘PD’ RILs) or our interspecific population generated by a cross between *C. annuum* and *C. chinense* (‘TH’ RILs). The 21 *C. frutescens* accessions used in the GWAS population may therefore not be sufficient to enable our detection of the *cap/cap7.2* locus in the present study.

We could compare the QTLs with the capsaicin‐associated SNPs detected from GWAS, but not with the dihydrocapsaicin‐ and total capsaicinoid‐associated SNPs, as their associations were not significant. From the raw GWAS results, nine and 42 SNPs were associated with the dihydrocapsaicin and total capsaicinoid contents, respectively. Among them, five and 36 SNPs were also detected as being associated with capsaicin content, and only these reached the FDR threshold for this association. Several GWAS and QTL mapping studies have demonstrated that one locus can control multiple highly correlated traits (Bauchet *et al*., 2017; Ben‐Chaim *et al*., 2006; Crowell *et al*., 2016; Han *et al*., 2016; Wang *et al*., 2011). We detected a high correlation between capsaicin, dihydrocapsaicin and total capsaicinoid contents; therefore, it is possible that some SNPs can affect these traits simultaneously. If the capsaicinoid contents of the GWAS population were evaluated repeatedly, we would expect to identify more significantly associated SNPs, enabling the validation of more QTLs using GWAS.

### Candidate genes controlling capsaicinoid content

Using a genome‐based approach, we found five candidate genes for controlling capsaicinoid contents in pepper: *pAMT*,* C4H*,* 4CL* and *CSE* from the phenylpropanoid pathway, and *FatA*, from the fatty acid pathway (Lang *et al*., 2009; Mazourek *et al*., 2009; Stewart *et al*., 2005). In plants, the phenylpropanoid pathway is known to be related to the biosynthesis of amino acids and diverse secondary metabolites (Fraser and Chapple, 2011; Vogt, 2010), and its involvement in the production of capsaicinoids was predicted based on intermediates and genes identified in other plant species (Curry *et al*., 1999; Mazourek *et al*., 2009). The expression of genes in the phenylpropanoid pathway was also found to be related to the capsaicinoid content of *Capsicum* fruits (Arce‐Rodriguez and Ochoa‐Alejo, 2017; Curry *et al*., 1999; Kim *et al*., 2009, 2014; Liu *et al*., 2012; Stewart *et al*., 2005; Zhang *et al*., 2016).

pAMT is the final enzyme of the phenylpropanoid pathway and mediates the formation of vanillylamine (Curry *et al*., 1999; Lang *et al*., 2009). The loss of *pAMT* function results in the biosynthesis of capsinoid, which has little to no pungency, rather than the pungent capsaicinoid (Jang *et al*., 2015; Lang *et al*., 2009; Tanaka *et al*., 2010a,b, 2015). In pungent peppers, *pAMT* was expressed only in the fruits, and its expression level in the developmental stages was positively correlated with that of other capsaicinoid biosynthesis genes, c*affeoyl‐CoA 3‐O‐methyltransferase*,* Kas*,* branched‐chain amino acid aminotransferase* and *Pun1*, as well as *C4H*, another candidate gene identified in our current study (Arce‐Rodriguez and Ochoa‐Alejo, 2017; Sarpras *et al*., 2016; Zhang *et al*., 2016). C4H functions at the endoplasmic reticulum to catalyse the reaction from cinnamate to coumarate (Mazourek *et al*., 2009), and another candidate gene, *4CL*, encodes the enzyme that acts immediately after C4H in this pathway (Mazourek *et al*., 2009). Another candidate gene, *CSE*, is located on chromosome 3 in a region that was detected in both the QTL mapping and the GWAS. CSE functions in the lignin biosynthetic pathway in the *Arabidopsis*,* Medicago*,* Populus* and *Panicum* genera, while in maize it has only a slight esterase activity (Ha *et al*., 2016; Vanholme *et al*., 2013). With the exception of this activity, its other functions in the Solanaceae family are unknown; therefore, further genetic studies are needed to elucidate its activities in *Capsicum*.

Compared with the phenylpropanoid pathway, not much is known about the fatty acid pathway because branched‐chain fatty acids are not common metabolites in most plant species outside of the Solanaceae family (Mazourek *et al*., 2009). FatA functions at the last stage of the fatty acid pathway and regulates the chain length of the fatty acids (Aluru *et al*., 2003). The expression of *FatA* during fruit maturation is correlated with capsaicinoid content (Aluru *et al*., 2003; Keyhaninejad *et al*., 2014; Zhang *et al*., 2016). Various transcription factors have been suggested to control the expression of the capsaicinoid biosynthesis genes, including *Erf*,* Jerf* and *CaMYB31* (Arce‐Rodriguez and Ochoa‐Alejo, 2017; Keyhaninejad *et al*., 2014); however, none of them were detected in our QTL and GWAS analyses.

The association of *pAMT* with pungency was detected only from the GWAS population, in which four *C. chinense* and fourteen *C. frutescens* accessions had a minor allele at SNP 3_26745367 that was linked to *pAMT* (data not shown). All accessions with minor alleles at SNPs linked to *C4H* and *CSE* were also *C. chinense*. In previous studies, *C. chinense* has been reported to have diverse nonfunctional alleles of *pAMT* affecting the levels of various capsaicinoids and capsinoids (Jang *et al*., 2015; Koeda *et al*., 2015; Tanaka *et al*., 2010a,b, 2015). Effects for *4CL* and *FatA* were detected only from the ‘TH’ RIL analysis in our study; even though no significant SNPs were identified in these regions from the GWAS, the capsaicinoid contents of plants with different alleles at these regions showed a varied distribution (Figure 4c). For *4CL*, the majority of the minor alleles were detected from *C. chinense* and *C. frutescens* accessions in the GWAS population. Species‐specific genetic variation can be used to breed highly pungent pepper cultivars by introgressing the candidate genes of *C. chinense* or *C. frutescens* into *C. annuum*.

### SNP detection by GBS for QTL study

We used the double‐digestion method to make GBS libraries for the ‘TH’ RIL and GWAS populations. PstI/MseI enzymes were used to digest both populations, with the additional use of EcoRI/MseI enzymes for the GWAS population. An *in silico* analysis revealed that approximately three times more effective cut sites (100–600 bp length fragments) were predicted when using the EcoRI/MseI enzymes to digest the ‘CM334’ reference genome than when PstI/MseI were used (data not shown), and that EcoRI/MseI made 30 times more cut sites in the regions where few SNPs were detected using PstI/MseI. The use of both sets of enzymes to construct GBS libraries therefore enabled the acquisition of sufficient SNPs for the GWAS.

The percentage of SNPs located in genic regions was highest in ‘TH’ RILs, and the SNP distribution graph revealed that they were concentrated in euchromatin regions (Table S11; Figure 2a). A similar biased distribution of SNPs was observed in soya bean GBS results generated using ApeKI (Sonah *et al*., 2013). Sonah and colleagues reported the ratio of SNPs located in soya bean genic regions was as high as 39.5%, which was very similar to the proportion in the ‘TH’ RIL population used PstI/MseI. Like ApeKI, PstI has partial methylation sensitivity in plants (Elshire *et al*., 2011; Pootakham *et al*., 2016; Truong *et al*., 2012), which could result in the identification of high SNP densities in genic regions. In the ‘PD’ RIL and GWAS populations, however, only 1.2% and 3.2% of SNPs, respectively, were located in genic regions. Of the two enzyme sets, libraries using EcoRI/MseI showed more number of SNPs than PstI/MseI in the GWAS population and might bring even distribution of SNPs. The large number of SNPs in our study demonstrates the effectiveness of using two enzyme sets for GBS. This approach could reduce the costs for genotyping and increase the number of effective SNPs in comparison with the use of one enzyme or one enzyme set.

In conclusion, we demonstrated that analysis using genome‐based QTL mapping and GWAS is a useful tool for the identification of candidate genes associated with capsaicinoid content, which was not easily achieved in previous studies using low‐density genetic maps. The candidate genes and their associated SNPs detected here will be helpful to improve our understanding of capsaicinoid biosynthesis and could be applied to the breeding of high‐pungency peppers. We also confirmed the minor effects of each locus and the epistatic effects between QTLs, revealing that multiple markers should be used together for marker‐assisted selection.

## Experimental procedures

### Plant materials

Two RIL populations were used in this study. The intraspecific population of 120 RILs (F_7:10_) was derived from *C. annuum* ‘Perennial’ × *C. annuum* ‘Dempsey’ (Han *et al*., 2016). Among them, 56 RILs were pungent and were used for the QTL analysis. An interspecific population of 85 RILs (F_9:11_) derived from *C. annuum* ‘TF68’ × *C. chinense* ‘Habanero’, provided by Prof. Byung‐Dong Kim of Seoul National University (Kim *et al*., 2010), was also used. These populations were referred to as ‘PD’ and ‘TH’ RILs, respectively, following their parental names. The ‘PD’ RIL population was grown in Hana Seed Co., Ltd., in Anseong (2011 and 2012a) and Seoul National University farm in Suwon, Republic of Korea (2012b), while ‘TH’ RILs were grown in Anseong (2013 and 2014). The plants in both Anseong and Suwon were grown in plastic greenhouses; however, the plants were grown in soil in Anseong and in pots in Suwon. Five plants were grown for each line.

A subpopulation of *Capsicum* core collections was used for GWAS (Lee *et al*., 2016a). To reduce the effect of the major gene controlling pungency, *Pun1*, all accessions were genotyped using the MAP1 marker (Rodríguez‐Maza *et al*., 2012; Stewart *et al*., 2005). A total of 208 accessions were selected, including 140 from the CC240 core collection (Lee *et al*., 2016a) and 68 additional accessions (Table S6). Five plants of each accession were grown in plastic greenhouses at the RDA‐GenBank in Jeonju, Republic of Korea.

### Evaluation of capsaicinoid content

The placental tissue of fruits from ‘PD’ RIL and ‘TH’ RIL was dissected for capsaicinoid extraction to reduce the effect of fruit size. Capsaicinoids were extracted following the method of Han *et al*. (2013). For the GWAS population, the modified protocol of Han *et al*. (2013) was used to extract the capsaicinoid. In short, three biological replicates were prepared by freeze‐drying whole fruits, which were then ground using a hand blender (HR2860; Koninklijke Philips, Amsterdam, the Netherlands) and stored in sealed containers at −80°C. To extract multiple samples at one time, 0.1 g of pepper powder was placed in a 2‐mL microcentrifuge tube and mixed with 1.5 mL of a 6:4 ethyl acetate:acetone solution. After incubation for 1 day at 37°C, 1 mL supernatant was transferred to a 1.5‐mL microcentrifuge tube and dried using a centrifugal speed vacuum concentrator SVQ‐70 (Operon, Gimpo, Republic of Korea). The pellet was dissolved in 1 mL methyl alcohol and filtered using a 0.2‐μm syringe filter (PN4450; Pall Corporation, Port Washington, NY).

The filtered extracts were transferred to a high‐performance liquid chromatography (HPLC) vial (5182‐0715; Agilent Technologies, Santa Clara, CA). The contents of capsaicin and dihydrocapsaicin were quantified using HPLC in the National Instrumentation Center for Environmental Management (Seoul, Republic of Korea). Capsaicin and dihydrocapsaicin standards were purchased from Sigma‐Aldrich (St. Louis, MO; M2028 and M1022, respectively).

### gDNA extraction and genotyping by sequencing

DNA was extracted from the ‘TH’ RIL and GWAS populations using the CTAB method (Lee *et al*., 2017) and diluted to 50 ng/μL with distilled water. GBS libraries of ‘TH RIL’ were generated by digestion with PstI/MseI using a SBG 100‐Kit v2.0 (Keygene N.V., Wageningen, the Netherlands), while those of the GWAS population were constructed manually following digestion with PstI/MseI and EcoRI/MseI, according to a previously reported protocol (Jo *et al*., 2017; Truong *et al*., 2012). In either case, DNA was digested with the restriction enzymes and adapters were ligated to it. The libraries were amplified with selective primers, which used ‘GA’ for ‘TH RIL’ and ‘TA’ for the GWAS population. Amplified libraries generated from 85 ‘TH’ RILs and two replicates of each of the population parents were pooled in a single tube. The libraries of the GWAS population were pooled in five tubes. All tubes were sequenced in separate lanes of a HiSeq 2000 (Illumina, San Diego, CA) at Macrogen (Seoul, Republic of Korea).

### Reference‐based SNP calling

Raw 101‐bp reads of the GWAS and ‘TH’ RIL libraries were trimmed to a minimum length of 80 bp and filtered for a minimum quality of Q20. The filtered reads were aligned to the *C. annuum* ‘CM334’ reference chromosomes v1.55 (Kim *et al*., 2014) using the Burrows‐Wheeler Aligner program v0.7.12 (Li and Durbin, 2010). For SNP calling and filtering, the GATK Unified Genotyper v3.3‐0 was used (DePristo *et al*., 2011). SNPs from the ‘TH’ RIL population were filtered for a minimum genotype quality of 20 and a minimum read depth of 3. For the GWAS population, SNPs were filtered for a minor allele frequency >0.03, a calling rate >0.6 and an inbreeding coefficient (F) >0.8.

### Bin map construction for the RILs

Missing data for parents were imputed using the FSFHapImputation plugin for Tassel 5 (Swarts *et al*., 2014), and the recombination breakpoints of the RILs were detected using a sliding window approach (Han *et al*., 2016; Huang *et al*., 2009). The ratio of SNPs with maternal and paternal genotypes was calculated for each window, defined as 18 linked SNPs, and the overall genotype of each window was decided. Ratios of >0.7, 0.3–0.7 and <0.3 were scored as maternal, heterozygous and paternal genotypes, respectively. With the exception of the threshold for the recombination breakpoints, the methods described by Huang *et al*. (2009) were used. The genetic locations of the bins were decided using the Carthagene program (De Givry *et al*., 2005).

The bin map of ‘PD’ RIL was constructed using SNPs from the re‐sequencing data and the sliding window approach (Han *et al*., 2016). The bin maps of both ‘PD RIL’ and ‘TH RIL’ were constructed based on the *C. annuum* ‘CM334’ reference genome (Kim *et al*., 2014) and were compared using physical locations on the reference genome by the Marker Browser Phyzen Genomics Institute (Seongnam, Republic of Korea) and the MapChart v2.2 program (Voorrips, 2002).

### QTL analysis for capsaicinoid content

QTLs controlling the contents of capsaicin, dihydrocapsaicin and total capsaicinoid (the combined capsaicin and dihydrocapsaicin contents) were independently detected for ‘PD RIL’ and ‘TH RIL’. Composite interval mapping was performed using Windows QTL Cartographer v2.5 (Wang *et al*., 2012), and the LOD threshold was determined by 500 permutation tests (*P *<* *0.05) for each trait. When the genetic locations of the QTLs (at a 99% significance level) overlapped in the plants grown in the different environments, they were defined as a single QTL. The physical locations of the QTLs from ‘PD RIL’ and ‘TH RIL’ were also compared with the genetic and physical location of bins linked to the QTLs. Epistatic effects between the QTLs were identified using a MIM analysis with a Bayesian information criterion (BIC‐X) model using the default options.

### Genomewide association analyses for capsaicinoid content

The 109 610 filtered SNPs detected from the 208 individuals of the GWAS population were used for association mapping. The population structure estimation (PCA and Kinship matrixes) and GWAS (based on the compressed mixed linear model) were conducted using the R package Genomic Association and Prediction Integrated Tool (Lipka *et al*., 2012) with default settings. The *P*‐values of SNPs from GWAS underwent an FDR analysis, and the FDR‐adjusted *P*‐value of 0.05 was used to set the significant threshold level.

### Haplotype block estimation and candidate gene identification

The haplotype block of the GWAS population was estimated using PLINK v1.9 (Chang *et al*., 2015) with the following settings: ‘–no‐parents –allow‐no‐sex –blocks‐max‐kb 2000 –blocks‐inform‐frac 0.9 –blocks‐strong‐highci 0.85 –blocks‐recomb‐highci 0.8’. Candidate genes located at the associated regions were identified, and their functions were annotated using Blast2Go (Gotz *et al*., 2008).

## Supporting information


**Figure S1** Correlation between the contents of capsaicin, dihydrocapsaicin, and total capsaicinoids in ‘PD’ RILs (a), ‘TH’ RILs (b), and the GWAS population (c). CAP, capsaicin; DICAP, dihydrocapsaicin; Total, total capsaicinoid.
**Figure S2** Comparison of the genetic maps of ‘PD’ and ‘TH’ RILs with the physical map. Bars on the left and right show the genetic map position (cM) and the physical map position (Mbp), respectively. PD, genetic map of ‘PD’ RILs; TH, genetic map of ‘TH’ RILs; CM334, physical map of the *C. annuum* ‘CM334’ reference genome.
**Figure S3** Population structure of the GWAS population, with a principal component analysis (a) and a phylogenetic tree (b) determined from 109 610 SNPs. Dark orange, blue and purple colours indicate *C. annuum*,* C. chinense* and *C. frutescens*, respectively.
**Table S1** Capsaicinoid contents (μg/g DW) of Perennial, Dempsey, and ‘PD’ RIL plants grown in three different environments.
**Table S2** Capsaicinoid contents (μg/g DW) of TF68, Habanero, and ‘TH’ RIL plants grown in two different environments.
**Table S3** Bin map of the ‘TH’ RIL population.
**Table S4** Genotypes of bins in the ‘TH’ RIL bin map.
**Table S5** Epistatic effects of major QTLs in ‘PD’ RILs.
**Table S6** Epistatic effects of major QTLs in ‘TH’ RILs.
**Table S7** Accessions used for GWAS.
**Table S8** Haplotype block estimated by genotyping by sequencing of the GWAS population.
**Table S9** Associated regions and candidate genes detected by GWAS.
**Table S10** Physical location of QTLs for validation.
**Table S11** Distribution of SNPs in genic and intergenic regions.
