# Supplementary material for: QTL mapping and GWAS reveal candidate genes controlling capsaicinoid content in Capsicum
Source: Plant Biotechnol J. 2018 Mar 2;16(9):1546–58. doi: 10.1111/pbi.12894 (PMC6097123; doi:10.1111/pbi.12894)
Supplement: Supplementary file 1 — Figure S1 Correlation between the contents of capsaicin, dihydrocapsaicin, and total capsaicinoids in ‘PD’ RILs (a), ‘TH’ RILs (b), and the GWAS population (c). CAP, capsaicin; DICAP, dihydrocapsaicin; Total, total capsaicinoid. Figure S2 Comparison of the genetic maps of ‘PD’ and ‘TH’ RILs with the physical map. Bars on the left and right show the genetic map position (cM) and the physical map position (Mbp), respectively. PD, genetic map of ‘PD’ RILs; TH, genetic map of ‘TH’ RILs; CM334, physical map of the C. annuum ‘CM334’ reference genome. Figure S3 Population structure of the GWAS population, with a principal component analysis (a) and a phylogenetic tree (b) determined from 109 610 SNPs. Dark orange, blue and purple colours indicate C. annuum, C. chinense and C. frutescens, respectively. Table S1 Capsaicinoid contents (μg/g DW) of Perennial, Dempsey, and ‘PD’ RIL plants grown in three different environments. Table S2 Capsaicinoid contents (μg/g DW) of TF68, Habanero, and ‘TH’ RIL plants grown in two different environments. Table S3 Bin map of the ‘TH’ RIL population. Table S4 Genotypes of bins in the ‘TH’ RIL bin map. Table S5 Epistatic effects of major QTLs in ‘PD’ RILs. Table S6 Epistatic effects of major QTLs in ‘TH’ RILs. Table S7 Accessions used for GWAS. Table S8 Haplotype block estimated by genotyping by sequencing of the GWAS population. Table S9 Associated regions and candidate genes detected by GWAS. Table S10 Physical location of QTLs for validation. Table S11 Distribution of SNPs in genic and intergenic regions. [file PBI-16-1546-s001.zip › pbi12894-sup-0001-Supinfo.docx]

**Table S1.** Capsaicinoid contents (μg/g DW) of Perennial, Dempsey, and ‘PD’ RIL plants grown in three different environments

|  | 2011 | | | | | 2012a | | | | | | | 2012b | | | | | | |
| --- | --- | --- | --- | --- | --- | --- | --- | --- | --- | --- | --- | --- | --- | --- | --- | --- | --- | --- | --- |
|  | Perennial | Dempsey | RIL | | | Perennial | | | Dempsey | RIL | | | Perennial | | | Dempsey | RIL | | |
| Capsaicin | 25,043 | ND | 9,675 | ± | 9,589.9 | 16,970 | ± | 1,368.7 | ND | 6,970 | ± | 6,846.4 | 42,793 | ± | 8,729.5 | ND | 11,812 | ± | 10,993.2 |
| Dihydrocapsaicin | 12,969 | ND | 6,880 | ± | 7,203.3 | 14,548 | ± | 1,439.3 | ND | 6,035 | ± | 6,381.5 | 38,463 | ± | 5,463.7 | ND | 10,449 | ± | 9,586.0 |
| Total capsaicinoid | 38,013 | ND | 16,555 | ± | 16,595.8 | 31,518 | ± | 2,808.0 | ND | 13,005 | ± | 13,159.0 | 81,257 | ± | 14,193.1 | ND | 22,058 | ± | 20,255.4 |
| ND, not detected | | | | | | | | | | | | | | | | | | | |

**Table S2.** Capsaicinoid contents (μg/g DW) of TF68, Habanero, and ‘TH’ RIL plants grown in two different environments

| Trait | 2013 | | | | | | | 2014 | | | | | | | | |
| --- | --- | --- | --- | --- | --- | --- | --- | --- | --- | --- | --- | --- | --- | --- | --- | --- |
|  | TF68 | | | Habanero | RIL | | | TF68 | | | Habanero | | | RIL | | |
| Capsaicin | 3,280 | ± | 1,585.8 | 50,484 | 12,646 | ± | 14,584.0 | 4,068 | ± | 4,067.8 | 38,102 | ± | 1,486.4 | 11,640 | ± | 11,317.0 |
| Dihydrocapsaicin | 2,392 | ± | 1,054.2 | 39,341 | 13,163 | ± | 15,531.4 | 3,131 | ± | 3,130.8 | 35,717 | ± | 2,607.3 | 12,314 | ± | 12,132.9 |
| Total capsaicinoid | 5,672 | ± | 2,572.7 | 89,825 | 25,809 | ± | 28,508.7 | 7,199 | ± | 7,198.6 | 73,819 | ± | 3,644.1 | 23,953 | ± | 22,595.7 |

**Table S3.** Bin map of the ‘TH’ RIL population

| Chr. | Number of SNPs | Number of bins | Physical length of bin (Mb) | | Genetic distance of bin (cM) | |
| --- | --- | --- | --- | --- | --- | --- |
|  |  |  | Mean | Total | Mean | Total |
| 1 | 920 | 118 | 2.3 | 272.7 | 1.3 | 155.2 |
| 2 | 804 | 93 | 1.8 | 171.1 | 1.2 | 112.1 |
| 3 | 1,060 | 127 | 2.0 | 257.9 | 0.9 | 111.7 |
| 4 | 657 | 102 | 2.2 | 222.6 | 0.8 | 81.7 |
| 5 | 672 | 105 | 2.2 | 233.5 | 0.7 | 78.2 |
| 6 | 794 | 101 | 2.3 | 236.9 | 0.9 | 89.9 |
| 7 | 679 | 78 | 3.0 | 231.9 | 1.0 | 80.5 |
| 8 | 541 | 35 | 4.1 | 145.1 | 1.1 | 39.6 |
| 9 | 614 | 88 | 2.9 | 252.8 | 1.0 | 88.5 |
| 10 | 589 | 83 | 2.8 | 233.6 | 1.3 | 105.4 |
| 11 | 613 | 88 | 3.0 | 259.7 | 1.0 | 87.3 |
| 12 | 644 | 71 | 3.3 | 235.7 | 1.4 | 97.2 |
| Total | 8,587 | 1,089 | 2.5 | 2,753.5 | 1.0 | 1,127.3 |

**Table S4**. Genotypes of bins in the ‘TH’ RIL bin map

Data is presented in the attached excel file.

**Table S5.** Epistatic effects of major QTLs in ‘PD’ RILs

| Trait | Year | QTL | R^2^ (%)* | Total R^2^ (%) |
| --- | --- | --- | --- | --- |
| Capsaicin content | 2011 | *PD-cap1* | 14.8 | 45.4 |
|  |  | *PD-cap10* | 21.8 |  |
|  |  | *PD-cap1*×*PD-cap10* | 8.8 |  |
| Dihydrocapsaicin content | 2012a | *PD-dicap2.1* | -0.4 | 17.5 |
|  |  | *PD-dicap10.2* | 10 |  |
|  |  | *PD-dicap2.1*×*PD-dicap10.2* | 7.9 |  |
|  | 2012b | *PD-dicap1.1* | 15.4 | 40.8 |
|  |  | *PD-dicap10.2* | 22.4 |  |
|  |  | *PD-dicap1.1*×*PD-dicap10.2* | 3.0 |  |
| Total capsaicinoid content | 2011 | *PD-total1.1* | 11.3 | 41.9 |
|  |  | *PD-total10.2* | 17.2 |  |
|  |  | *PD-total1.1*×*PD-total10.2* | 13.4 |  |
| *R^2^ value of individual QTL and interactions of QTLs were evaluated by MIM analyses | | | | |

**Table S6**. Epistatic effects of major QTLs in ‘TH’ RILs

| Trait | Year | QTL | R^2^ (%)* | Total R^2^ (%) |
| --- | --- | --- | --- | --- |
| Capsaicin content | 2014 | *TH-cap2.2* | 13.7 | 20.4 |
|  |  | *TH-cap4* | 5.3 |  |
|  |  | *TH-cap2.2*×*TH-cap4* | 1.4 |  |
| Total capsaicinoid content | 2014 | *TH-total2* | 37.3 | 57.2 |
|  |  | *TH-total3.2* | -0.4 |  |
|  |  | *TH-total10* | 18.5 |  |
|  |  | *TH-total2*×*TH-total3.2* | -0.6 |  |
|  |  | *TH-total2*×*TH-total10* | 2.3 |  |
|  |  | *TH-total3.2*×*TH-total10* | 0.1 |  |
| *R^2^ value of individual QTL and interactions of QTLs were evaluated by MIM analyses | | | | |

**Table S7.** Accessions used for GWAS

| Species | Number of accessions | | |
| --- | --- | --- | --- |
|  | CC240* | Additional accessions | Total |
| *Capsicum annuum* | 110 | 35 | 145 |
| *Capsicum chinense* | 16 | 26 | 42 |
| *Capsicum frutescens* | 14 | 7 | 21 |
| Total | 140 | 68 | 208 |
| *Lee et al., 2016a | | | |

**Table S8.** Haplotype block estimated by genotyping-by-sequencing of the GWAS population

Data is presented in the attached excel file.

**Table S9**. Associated regions and candidate genes detected by GWAS

Data is presented in the attached excel file.

**Table S10**. Physical location of QTLs for validation

Data is presented in the attached excel file.

**Table S11**. Distribution of SNPs in genic and intergenic regions

| Population | Number of SNPs (%) | | |
| --- | --- | --- | --- |
|  | Genic | Intergenic | Total |
| PD RIL | 16,581 (1.2) | 1,414,633 (98.8) | 1,431,214 |
| TH RIL | 3,307 (38.5) | 5,280 (61.5) | 8,587 |
| GWAS population | 3,453 (3.2) | 106,157 (96.8) | 109,610 |

**
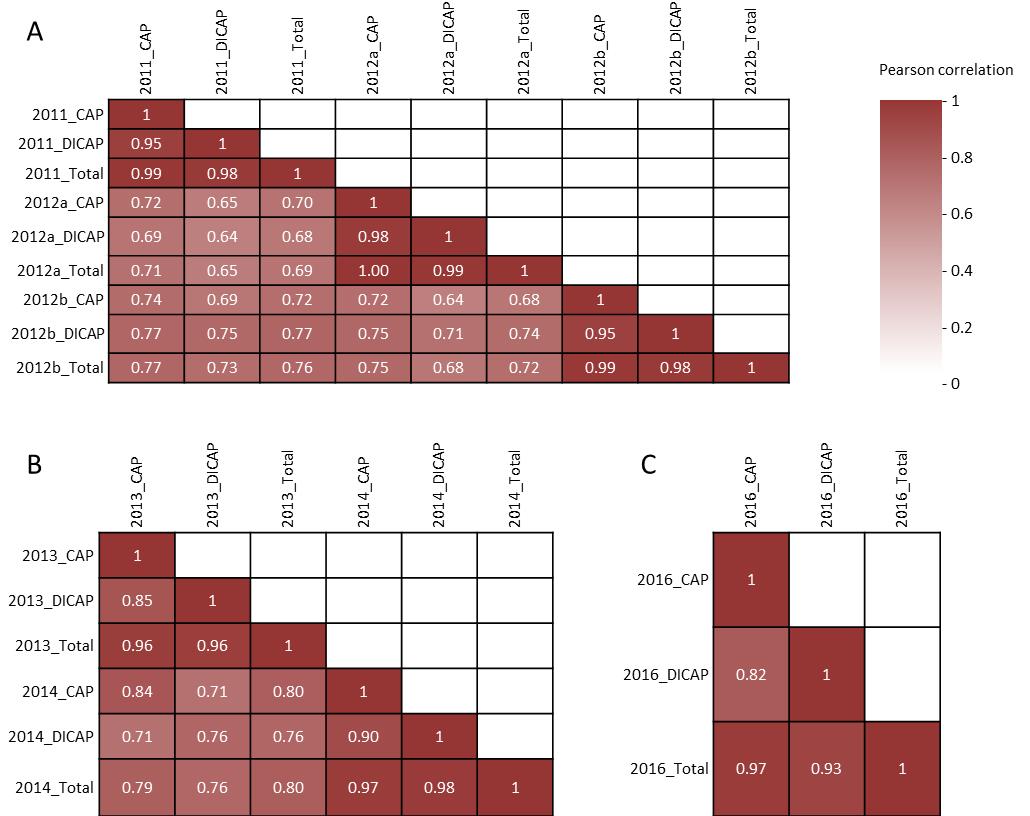
**

**Figure S1.** Correlation between the contents of capsaicin, dihydrocapsaicin, and total capsaicinoids in ‘PD’ RILs (A), ‘TH’ RILs (B), and the GWAS population (C). CAP, capsaicin; DICAP, dihydrocapsaicin; Total, total capsaicinoid.


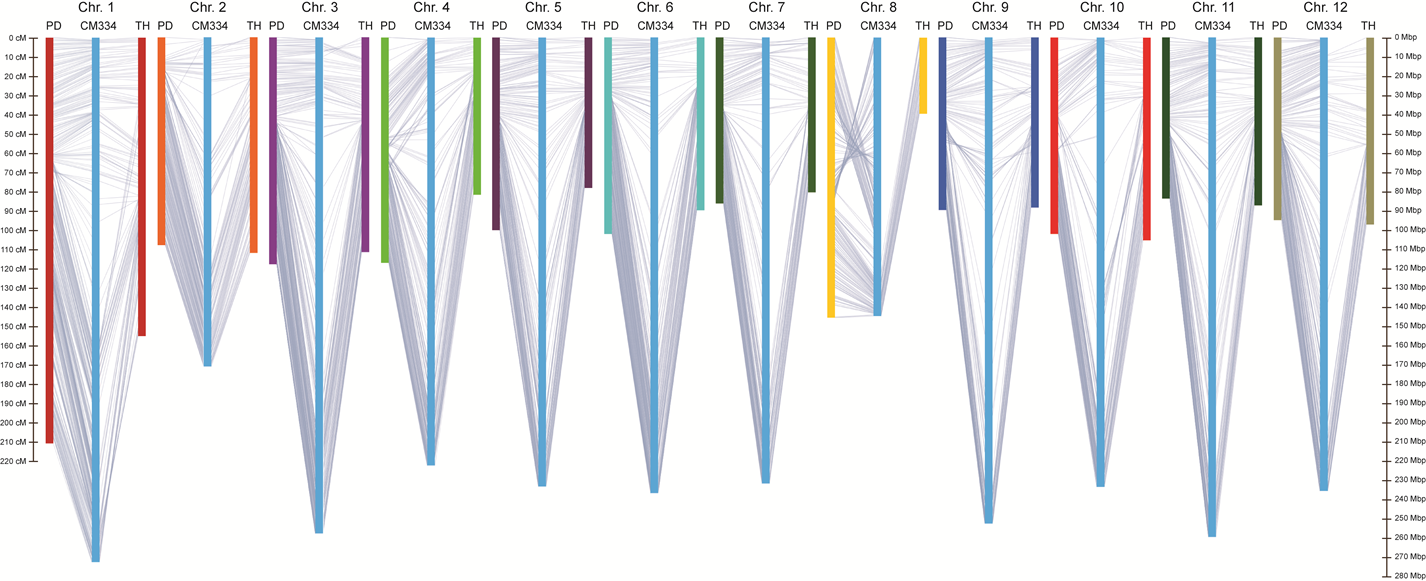


**Figure S2.** Comparison of the genetic maps of ‘PD’ and ‘TH’ RILs with the physical map. Bars on the left and right show the genetic map position (cM) and the physical map position (Mbp), respectively. PD, genetic map of ‘PD’ RILs; TH, genetic map of ‘TH’ RILs; CM334, physical map of the *C. annuum* ‘CM334’ reference genome.

**
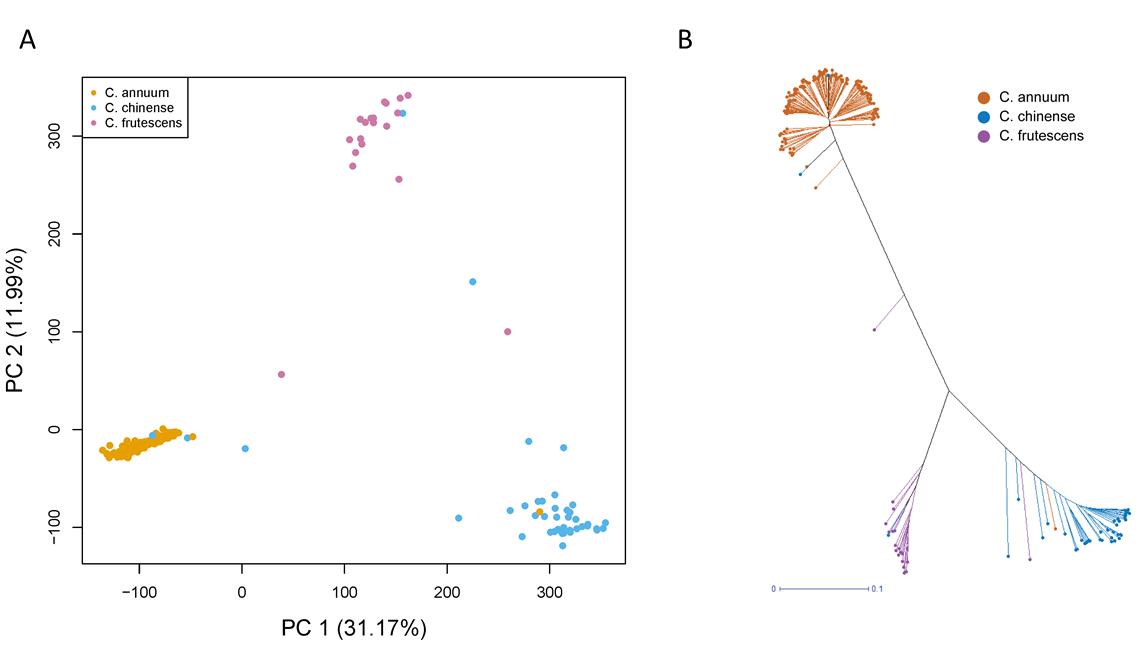
**

**Figure S3.** Population structure of the GWAS population, with a principal component analysis (A) and a phylogenetic tree (B) determined from 109,610 SNPs. Dark orange, blue, and purple colors indicate *C. annuum*, *C. chinense*, and *C. frutescens*, respectively.
